# A dynamic fuzzy rule-based inference system using fuzzy inference with semantic reasoning

**DOI:** 10.1038/s41598-024-54065-1

**Published:** 2024-02-21

**Authors:** Nora Shoaip, Shaker El-Sappagh, Tamer Abuhmed, Mohammed Elmogy

**Affiliations:** 1 Information Systems Department, Faculty of Computers and Information, Damanhour University, 22511 Damanhour, Egypt; 2 Faculty of Computer Science and Engineering, Galala University, Suez, 435611 Egypt; 3 Information Systems Department, Faculty of Computers and Artificial Intelligence, Benha University, Banha, 13518 Egypt; 4 Department of Computer Science and Engineering, College of Computing and Informatics, Sungkyunkwan University, Seoul, Republic of Korea; 5 Information Technology Department, Faculty of Computers and Information, Mansoura University, Mansoura, 35516 Egypt

**Keywords:** Alzheimer’s disease, Fuzzy rule-based systems, Clinical decision support system, Semantic similarity, Ontology reasoning, Electrical and electronic engineering, Health care

## Abstract

The challenge of making flexible, standard, and early medical diagnoses is significant. However, some limitations are not fully overcome. First, the diagnosis rules established by medical experts or learned from a trained dataset prove static and too general. It leads to decisions that lack adaptive flexibility when finding new circumstances. Secondly, medical terminological interoperability is highly critical. It increases realism and medical progress and avoids isolated systems and the difficulty of data exchange, analysis, and interpretation. Third, criteria for diagnosis are often heterogeneous and changeable. It includes symptoms, patient history, demographic, treatment, genetics, biochemistry, and imaging. Symptoms represent a high-impact indicator for early detection. It is important that we deal with these symptoms differently, which have a great relationship with semantics, vary widely, and have linguistic information. This negatively affects early diagnosis decision-making. Depending on the circumstances, the diagnosis is made solo on imaging and some medical tests. In this case, although the accuracy of the diagnosis is very high, can these decisions be considered an early diagnosis or prove the condition is deteriorating? Our contribution in this paper is to present a real medical diagnostic system based on semantics, fuzzy, and dynamic decision rules. We attempt to integrate ontology semantics reasoning and fuzzy inference. It promotes fuzzy reasoning and handles knowledge representation problems. In complications and symptoms, ontological semantic reasoning improves the process of evaluating rules in terms of interpretability, dynamism, and intelligence. A real-world case study, ADNI, is presented involving the field of Alzheimer’s disease (AD). The proposed system has indicated the possibility of the system to diagnose AD with an accuracy of 97.2%, 95.4%, 94.8%, 93.1%, and 96.3% for AD, LMCI, EMCI, SMC, and CN respectively.

## Introduction

Alzheimer’s disease (AD)^1^, as a progressive mental and neurological disorder, was discovered in 1906 when Dr. Alois Alzheimer observed and found numerous amyloid plaques and tangled bundles of tau proteins in the brain of a deceased woman. She was suffering from language problems and memory loss. The main feature of AD is that the accumulation of these plaques in the brain causes the loss of connections between nerve cells. AD causes the loss of memory, reasoning, and cognitive functions, and all sense of self seems to disappear^2^. As AD progresses, dementia usually follows, and patients cannot communicate effectively and are completely dependent on others for care. AD harms societies and continues to threaten the lives of billions of elderly^3^.

The symptoms of AD progressively deteriorate over time^4^. Initially, patients show a decline in cognitive functions, characterized by difficulties in learning new information and an increased tendency to observe their surroundings. As the disease progresses, patients experience moderate cognitive impairments, which manifest as repetitive questioning, difficulties in managing finances and settling bills, coping with unfamiliar situations, and recognizing relatives and friends. Finally, AD advances to affect critical physiological activities, including motor coordination and essential functions such as respiration and swallowing^5^. Early detection of AD is a critical task for the practicing physician. Despite many medical and cognitive tests’ availability, the hoped-for detection efficiency was not achieved^6^. It is usually due to neglect of cognition and confusion with normal aging. AD is multimodal; its detection requires the patient’s symptoms, genetic history, medications, and different imaging, psychological and neurological tests. These tests can help detect damage or atrophy in some parts of the brain. And thus determine the degree of risk that the patient has reached^7^.

As a method to enable knowledge representation, reasoning tasks, and the sharing of knowledge among individuals, software agents, and systems^8^, Ontology serves as a crucial component that enables the creation of machine-processable structures to enrich knowledge reasoning within specific fields^9^. The Ontology Language (OWL) is used for constructing ontologies and has two versions: OWL1 and OWL2^10^. In the medical domain, ontologies are employed in various areas such as anatomy, genetics, and more. Numerous Alzheimer’s disease (AD) ontologies have been previously developed, including SWAN, MIND, ND, Ontology-Driven Decision, OntoNeuroLOG, Multiagen, ADO, and ADMO. While most of these ontologies facilitate the representation, storage, and retrieval of AD-related information, only a few support AD diagnosis. Their computational reasoning capabilities for inference remain limited and do not comprehensively address various diagnostic aspects. Reviews of these ontologies can be found in^11,12^.

Medical data can be characterized by three primary attributes: categorical, numerical, and textual^13^. Categorical attributes (e.g., smoking and alcohol consumption) can be represented as a set of predetermined values, often limited to binary options such as yes or no. Numerical attributes typically consist of data from cognitive, psychological, and laboratory tests, as well as measurements derived from imaging, like dimensions of specific brain regions. Both categorical and numerical attributes can be conveniently treated as fuzzy linguistic variables.

**Figure 1:**
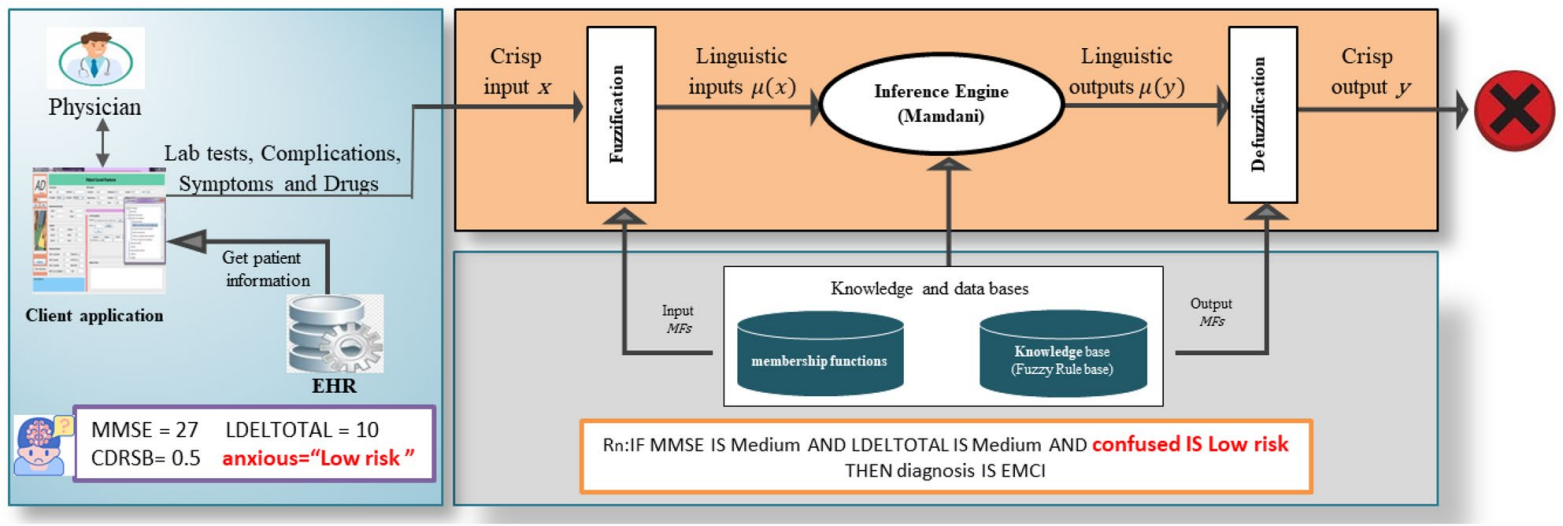
A static fuzzy rule-based reasoning system. Incorrect handling of semantic data may have a negative impact on the inference process. As it requires flexibility to deal with synonyms. For example, this figure shows an inference problem. The reason for this is that regular static rules can only search for specific features with specific values. In this case, the antecedent of a static rule contains MMSE, LDELTOTAL, and confusion. The confusion feature is semantic data related to symptoms of mood and personality changes that can be expressed with many synonyms. When the user introduces another synonym, such as doubt, depression, or fear, the static rule will not be able to understand that those terms may also mean confusion.

However, textual attributes, which encompass patient complications and symptoms, present a distinct challenge. Complications and symptoms are rich in synonyms and terms that ultimately lead to the same meaning. It is inappropriate to treat them as categorical variables with fixed values, as they consist of a diverse range of terminologies that can vary among patients. For instance, in a static-rule-based fuzzy reasoning system (see Fig. 1), if there is a static rule concerning symptoms of mood and personality change with the conditional constant “confused” defined by a low-risk value, the system would be unable to comprehend that terms like suspicious, depressed, fearful, or anxious could also imply confusion. Thus, employing ontology provides a more dynamic and intelligent approach, as it can discern similarities and differences of each term related to mood and personality changes with respect to its neighbors: its parent class and its siblings. It can understood that anxiety and confusion are close in meaning.

Ontology is defined as a formal representation of knowledge in a certain domain. It consists of defined classes of entities, typically structured within a knowledge hierarchy where concepts are connected by standardized semantic relationships (i.e., “is-a,” “part-of”). It serves as a means to standardize concepts by establishing a structured framework for terms and their relationships, which describe the underlying processes. Through semantic interoperability, ontologies enable the meaningful exchange of information among vast medical data resources^14^. The development of an ontology often requires significant time and effort in order to create consistent and reusable ontologies^15^. provides an overview of the ontology design methodology. The initial step involves confirming the domain and scope of the ontology, identifying its key aspects, and deciding whether to construct it from scratch or reuse an existing ontology. The second step entails identifying current knowledge sources and gathering information through surveys and reviews of existing ontologies. The third step, ontology initialization and merging, involves defining the ontology’s classes, properties, and instances and merging the top-level ontology with the mid-level ontology. This process situates the ontology as a sub-ontology of the Basic Formal Ontology (BFO)^16^, the Ontology for General Medical Science (OGMS) top-level ontologies^17^, and standard terminologies. The fourth step comprises implementing the ontology modules defined during the previous stage using an ontology editor. The final step involves testing and validating the ontology to ensure its accuracy and consistency, employing tools such as ontology reasoners for this purpose.

While ontology is a powerful and essential tool for representing medical knowledge, it has limitations. It is not well-suited for handling ambiguous data, which is frequently encountered in medical science due to the inherent uncertainty and inaccuracy^18,19^. For example, (1) concentrations of tau and beta-amyloid in cerebrospinal fluid may be reported as weak, moderate, or strong, (2) cognitive test results, such as those from the CDR, MMSE, or ADAS-Cog, can be classified as normal or abnormal, while (3) APOE4 test results may be described as negative, weakly positive, or positive. These examples illustrate the considerable degree of ambiguity in the medical domain. Ambiguity is formally characterized by the absence of precise boundaries. To address this issue, there is a need for ontology to be capable of representing and inferring information that is imprecise or ambiguous. This can be achieved by extending ontology with concepts derived from fuzzy set theory, which is specifically designed to deal with such uncertainties.

There are already several attempts to integrate ontology and fuzzy logic, among which we mention the following. First, the implementation of a particular fuzzy OWL ontology is proposed^20^. But still, there is no standard fuzzy OWL under the World Wide Web Consortium (W3C)^21^. Thus, fuzzy OWL is still not enough to model real applications. Second, the extension happens with fuzzy concepts and fuzzy rules^22^ and using FuzzyJess APIs and fuzzy logic to reason over it. However, the capabilities of fuzzy logic reasoning are limited because FuzzyJess has no plugin in Protégé and no suitable ontology reasoners can handle the fuzzy extension well. Third, FuzzyOWL2ontology, proposed by Bobillo and Straccia^23^, uses a regular crisp ontology editor. In this ontology, the fuzzification elements are added to the ontology using annotations and translated by parsers into a language usable by a fuzzy DL reasoner (e.g., Delorean is translating fuzzy ontologies into its crisp DLs and uses DLs reasoners as Pellet). However, existing ontology parsers and reasoners have not reached a mature state yet^24^. The knowledge-based applications, especially medical ones, are still somewhat weak, perhaps because they have not yet reached the state of reality. This is a problem for all fuzzy rule-based or ontology-based applications^25^. For fuzzy rule-based, facing textual features such as symptoms is a problem. Dealing with uncertainty and fuzzification remains an ontology problem.

In this paper, we provide one possible solution to these limitations. We proposed a dynamic fuzzy rule-based inference for estimating the AD diagnosis. It is an enrichment of classical fuzzy rule-based with ontology reasoning. It contributes to unique improvements in the rules’ evaluation process of fuzzy rule-based in terms of interpretability, dynamism, and intelligence. Semantic features, such as symptoms, complications, and drugs, are represented in the form of an ontology. The proposed ontology makes it possible to measure the clinical similarity between patient semantic features and their concepts, then reason on the severity of these features to be or not added in the final fuzzy rules evaluations according to the used membership functions (MFs).

The rest of the paper is organized as follows: in the next section, we discuss research efforts directed toward developing an AD diagnostic system. Below is a brief description of the ADNI data set and its motivations. Next, our methodology for the diagnosis of AD based on a dynamic fuzzy rule-based inferences system (DFRI) and their building blocks will be explained. Then, the experimental results are presented. Finally, we discuss our conclusions and future work.

## Related works

Early diagnosis systems for AD have become an active research area. Artificial intelligence branches have revolutionized the field of AD detection, including knowledge representation, machine learning (ML), deep learning (DL), and standard datasets. We have combined recent AD research (e.g., ML- and DL-based or ontology-based research), focusing on their methods and limitations to research the best detection techniques and the impact of using ontology.

### DL-based researches

In recent years, the use of DL methods in diagnosing AD has increased significantly, and there is no doubt that it has achieved results with high accuracy. Table 1 provides a brief overview of some of the recently published articles related to DL/AD algorithms

**Table 1:** An overview of some of the recently published articles related to DL/AD algorithms.

Author	Biomarker	Dataset	Methodology	Classifier	Accuracy
Samhan et al.^26^ (2022)	scans of the brain (CT, MRI,and X-ray)	Kaggle contains a set of 10432 images	a DL model	Convolutional Neural Networks CNN	100%
Chui et al.^27^ (2022)	common MRI scans	three benchmark OASIS datasets (434 samples for OASIS-1, 373 samples for OASIS-2, and 2168 samples for OASIS-3)	A three-tier algorithm comprised of Generative adversarial network (GAN), CNN, and, transfer learning (TL)	GAN-CNN models	96.9 %for OASIS-1, 96.1% for OASIS-2, and 97.5 % for OASIS-3.
Liu et al.^28^ (2021)	MRI	OASIS MRI dataset in addition, MRI scans were utilized from the ADNI.	Combining The depthwise separable convolution (DSC) and CNN. Moreover, transfer learning (GoogLeNet and AlexNet) is employed.	depth-wise separable CNN algorithm	AlexNet and GoogLeNet got 91.40% and 93.02% respectively
Savaş et al.^29^ (2022)	MRI	ADNI (2182 subjects)	Different models using CNN architecture	CNN algorithm	EfficientNetB0 got 92.98%.
El-Sappagh et al.^30^ (2020)	MRI, PET, CSD, ASD, NPD	ADNI (1536 subjects)	An ensemble multimodal multitask deep learning model	Combination of CNN and a bidirectional long short-term memory (BiLSTM)	92.62%
El-Sappagh et al.^31^ (2021)	MRI, PET, lab tests, neuropsychological test, cognitive test, demographics, and genetics	ADNI (1048 subjects)	AForest-based interpretable AD detection and progression prediction model.	a two-layer model with random forest (RF) as classifier algorithm	93.95%

They have primarily focused on achieving high accuracy in diagnosing AD. It is mainly based on neuroimaging data, especially MRI.

Although efficiency is essential in the AD diagnoses system, DL/AD algorithms neglect several important factors as follows.

- The challenging nature of the AD, as it is multimodal. It is related to symptoms, demographics, medical history, cognitive scores, neurology vital signs, neuropsychiatric problems, lab tests, etc. Therefore, a reliable medical diagnosis should not be based solely on unique field measurements.
- Issues of standardization, interpretation, maintaining interoperability, serving queries, explaining diagnostic decisions, etc., which are very well achievable in terms of semantic models.

### Ontology-based researches

Much attention has been paid to the knowledge representation of AD in order to create a consistent, modular, and reusable ontology. To support standardization, store and retrieve knowledge, and aid in the diagnosis of AD. Sanchez et al.^32^ proposed MIND ontology, which constitutes an ontology-based diagnostic system for early AD detection. It relied on various diagnostic tests, including neurological, radiological, neuropsychological, genetic, and metabolomics tests. It is a semantic reasoning system that uses certain facts and axioms. However, the MIND ontology is not publicly available. Zhang et al.^33^ suggested an ontology-driven decision support system. This system is to efficiently assist clinicians in the clinical diagnosis of MCI. Dependence of cortex thickness in clinical settings to classify CN and MCI patients, using an ontology set and rules generated by the C4.5 algorithm based on training data of 187 selected MCI and 177 normal (ADNI) patients. But the rule set used is static, while it is convenient to dynamically update the rule set. It also focuses on an MRI imaging approach.

Ivascu et al.^34^ developed an ontology-based multi-agent as a symptom-sensor-disease system for patients at risk for cognitive impairment. It provides real-time information to facilitate remote monitoring of patients. This work combines ontology with a multi-agent system to issue a diagnosis. The ontology is used as active knowledge to describe the Diseases, Conditions, signs, symptoms, and Sensors concepts. When a patient exhibits certain symptoms of a particular disease, that data is received from sensors. Then, the collected data is transmitted to the cloud, where the diagnosis can be determined. However, there are many challenges related to remote patient monitoring and data privacy improvements.

Sanchez et al.^32^ suggested a knowledge-based that relies on a semantic approach for AD diagnosis. The reasoning process that leads is driven by a set of fixed rules. This system was then developed by TORO et al.^35^ so that it enables the discovery of new rules that drive reasoning. It is based on the experience learned from the user and the reasoning capabilities offered by ontologies. Shoaip et al.^36^ established OWL/SWRL semantic reasoning model that exploited the ADNI dataset and machine learning (ML) techniques. This model expands the AD knowledge base reasoning ability by effective inference rules. It was able to define the current status of AD patients with an accuracy of 94.1%. However, the reasoning ignored symptoms and patient disease history and is based on crisp rule-based. Shoaip et al.^37^ developed ADDO ontology, a standard based on fuzzy ontology, and ADDO fully adheres to BFO and OGMS. It supports the clinical diagnosis of AD based on the representation of various aspects of patients, including MRI, complications, disease, physical examination, medical history, symptoms, laboratory examination, and genetics. Protégé was used to build ADDO, and then the extended Fuzzy was done using the FuzzyOWL2 plugin. It is one of the most important vague semantic knowledge, but it is huge and complex. A neural network-based deep neural network (CNN) was proposed by Bangyal et al.^38^, who have achieved better results when building ontology with the help of deep learning knowledge. The AD data set used in the experiment was obtained from Kaggle. Compared with machine learning-based methods (gradient boosting, logistic regression, SGD, MLP, XGB, random forest, SVM, KNN), CNN achieved the highest accuracy of 94.61%.

Despite current research efforts, the detection and prediction of AD remain a difficult problem. It requires flexibility to be able to determine appropriate diagnoses for any given set of conditions and to deal with many diagnostic categories with the varying nature of their data types. This can be achieved by relying on a dynamic fuzzy rules-based system that can deal efficiently with semantic data such as symptoms. Therefore, in this paper, we develop and implement the integration of fuzzy inference and ontology reasoning, resulting in DFRIs.

## Dataset

Patient records are from the Alzheimer’s Disease Neuroimaging Initiative (ADNI) database (adni.loni.usc.edu). It is one of the most common benchmark data in the field of AD. With great effort and multi-million dollar investment, the ADNI database has been established for aged citizens from the USA and Canada, including those with normal cognitive abilities, MCI, and Alzheimer’s patients aged 55–90 years. Through demographics, vital signs, medical history, and neuropsychological assessment combined with magnetic resonance imaging and positron emission tomography, ADNI provides a measure of the longitudinal progression of AD. It contributes to the possibility of early diagnosis.

Eligible Patients were selected from different stages of ADNI (e.g., ADNI-1, ADNI-2, ADNI-GO, and ADNI-3). Longitudinal data were not considered in this research, and the main reliance was on baseline visits only. Subjects are divided according to their baseline diagnosis into five categories [normal cognitive (CN), significant memory concern (SMC), early mild cognitive impairment (EMCI), late mild cognitive impairment (LMCI), and AD). Our data set contains 2268 subjects (47% female), ranging in age from 55 to 91 years. In all, 518 subjects were diagnosed as having CN; 313 were SMC subjects; 389 have been the subject of EMCI; 651 of them were LMCI subjects; 397 were AD subjects.

**Figure 2:**
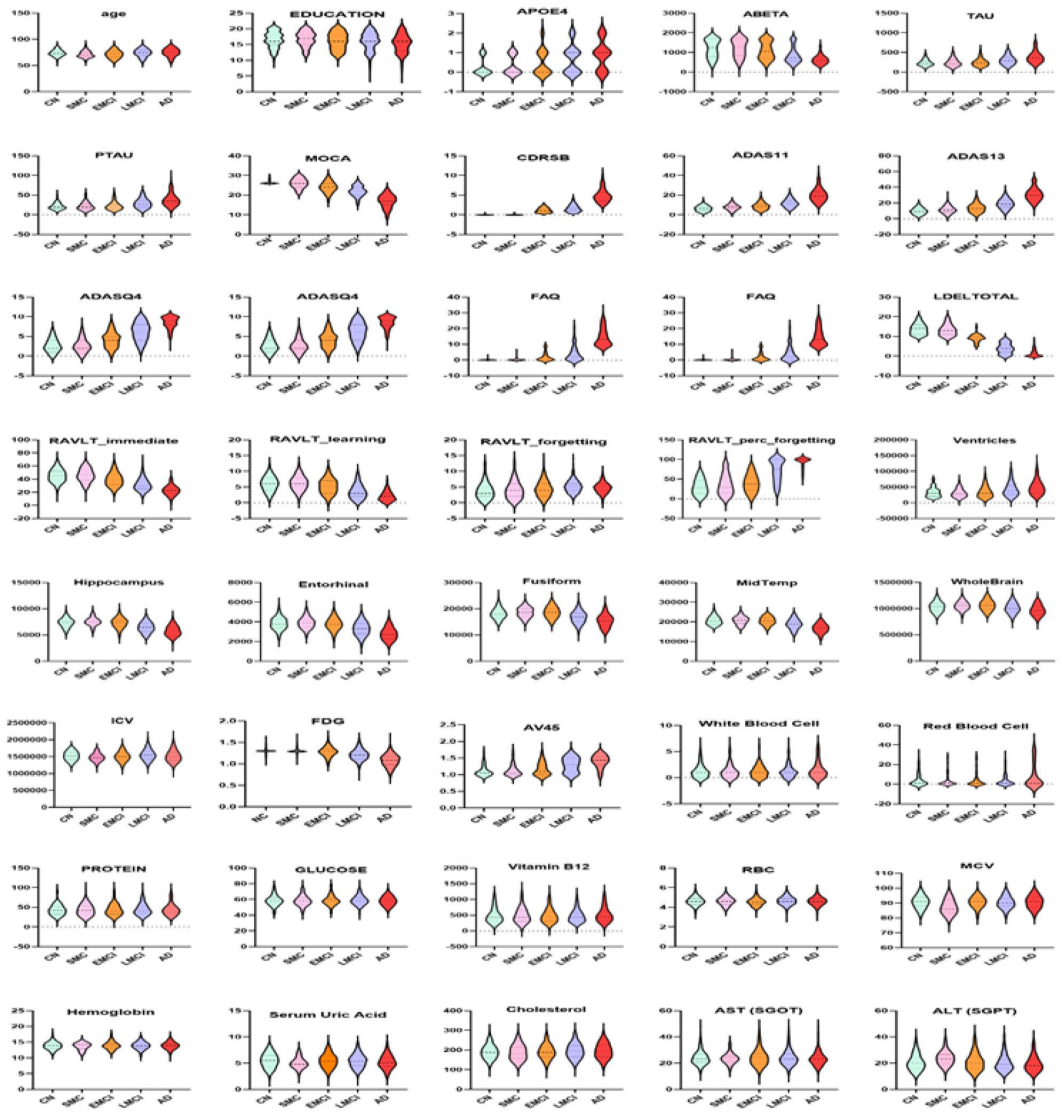
An analysis of some biomarkers of the ADNI dataset and their mean values by violin plot. As a result, we found that a high value of FAQ and CDRSB will increase the probability of detecting an AD patient. While a high MMSE and MOCA are more characteristic of the CN category. As for the large values LDELTOTAL, RAVLT_perc_forgetting, ADAS 11, ADAS 13, ventricles, TAU, and PTAU positively affect the prediction of the LMCI category. Small values of the FDG, MidTemp, and hippocampus predict EMCI.

AD has heterogeneous biomarker modalities. On the ADNI numerical sample data, we considered features associated with AD like demographics, vital signs, genetics, cognitive scores, lab tests, MRI, and PET. We described the full biomarker distribution by violin plot. It is a useful statistical method when plotting numerical data. As shown in Fig. 2, it is able to show which features have the greatest effect in revealing the degree of severity of AD.

AD may be associated with demographic factors such as age and education level. Age (< 65 years) is a demographic that distinguishes CN cases, while significantly fewer years of education distinguishes AD subjects. For genetic factors, the ε 4 alleles of the aneuploidy protein E (APOE4) gene are significant. APOE4 value tends to increase the chances of developing AD. Cerebrospinal fluid (CSF) may be an effective biomarker for detecting neuronal damage in AD. Biochemical changes of CSF proteins, including amyloid $\beta$ ta 1–42 (A$\beta$ 42), P-tau, and T-tau, are reflected in cognitive status^39^.

Early measurement of behavioral, functional, and psychosocial changes ensures early diagnosis of AD. The ADNI database covers a wide range of the most commonly used cognitive assessment tests. ADNI included: Montreal Cognitive Assessment (MoCA)^40^; Clinical Dementia classification Sum of Boxes (CDRSB)^41^; Mini-Mental State Examination (MMSE)^42^; Alzheimer’s Disease Assessment Scale 13 (ADAS13)^43^; Functional Assessment Questionnaire (FAQ)^44^; and Rey Auditory Verbal Learning Test (RAVLT)^45^; Logical Memory-Delayed Recall (LDELTOTAL)^46^; Immediate, Learning, Forgetting, and Perc Forgetting. Cognitive scores have a significant role in detecting AD patients.

A few brain instances of problems, such as ventricular enlargement and hippocampal atrophy, have been associated with normal aging, but AD has the most severe effect on brain structure. Aging affects the brain independent of AD pathology. Neuroimaging measures utilized Brain MRI (magnetic resonance imaging) and PET data. Brain MRI is frequently used to monitor and evaluate brain tissue’s existing volume and position. The ADNI MRI features were processed by a team from UCSF, who used FreeSurfer version 6.0 image analysis and performed volumetric segmentations. It included volumetric data of the entorhinal, fusiform gyrus, hippocampus, ventricles, middle temporal gyrus (MidTemp), intracerebral volume (ICV), and whole brain. Brain MRI^47^ is an important diagnostic tool capable of predicting progress. It is painless and doesn’t use radiation. Mainly, it uses a computer, radio waves, and a large magnet to produce these detailed images. MRI of the brain is often done based on the presence of certain symptoms or to monitor diagnosed patients. This highlights the importance of managing symptoms as an important early detection point. The significant criteria for AD are the accumulation of amyloid-beta plaques and decreased cortical glucose metabolism. An imaging technique that can be used to measure these criteria is fluorodeoxyglucose positron emission tomography (FDG PET)^48^. It shows metabolism in the brain by measuring glucose levels.

## System methodology

**Figure 3:**
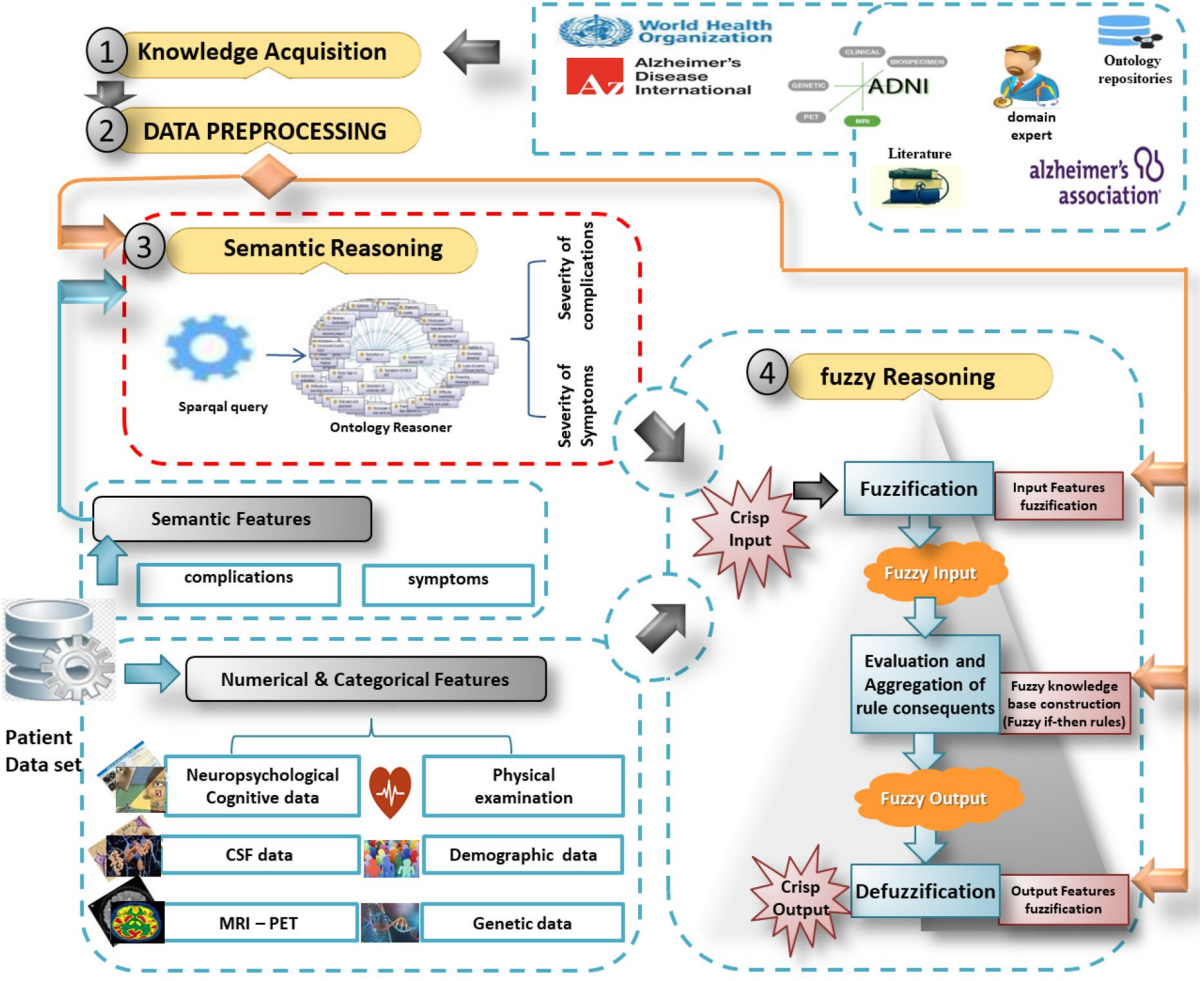
The proposed DFRIs structure. The main goal of DFRIs is the early and accurate diagnosis of AD. It is characterized by the unique handling of semantic data such as symptoms. This is due to the use of ontology reasoning, which allows receiving symptoms without restricting them to a specific number or values, then calculating the severity of these symptoms and expressing it with numerical data that can be sent to Fuzzy Logic along with other diagnostic data (i.e., lab test, cognitive tests, etc.). Where it is easy to treat these numerical features as fuzzy linguistic variables and get inference diagnosis results.

To provide semantically intelligent improvements in the process of evaluating fuzzy rules, we propose DFRIs. It’s a fuzzy system integrated with a semantic ontology. The proposed methodology has many blocks. As shown in Fig. 3, it includes knowledge acquisition, data processing, semantic reasoning, fuzzy inference, system implementation, and system testing. Each of these blocks will be explained as follows.

### Knowledge acquisition

Acquisition of knowledge is one of the most important parts of developing a fuzzy reasoning system for medical diagnosis. Acquiring knowledge in an integrated and accurate manner helps diagnose the disease efficiently. This study gathers knowledge about AD from several ways, including searching some of the websites that support and conduct Alzheimer’s research [(World Health Organization (WHO), Alzheimer’s Association, National Institute on Aging (NIA)], Medical Experts, recent literature, and ADNI electronic health record databases.

### Data preprocessing

Data is usually obtained from various sources using data mining techniques in the real medical field. It may be corrupted by noise, inconsistency, and missing values, leading to wrong decisions. Implementing a set of pre-processing steps improves the quality of medical data collected. Data Quality involves main activities, including removing outliers, encoding categorical features, smoothing the noisy data, and integrating data to merge the present data into larger data. According to Medical Concepts, semantic encoding is preferred according to the SCT standard terminology.

### Semantic reasoning

**Figure 4:**
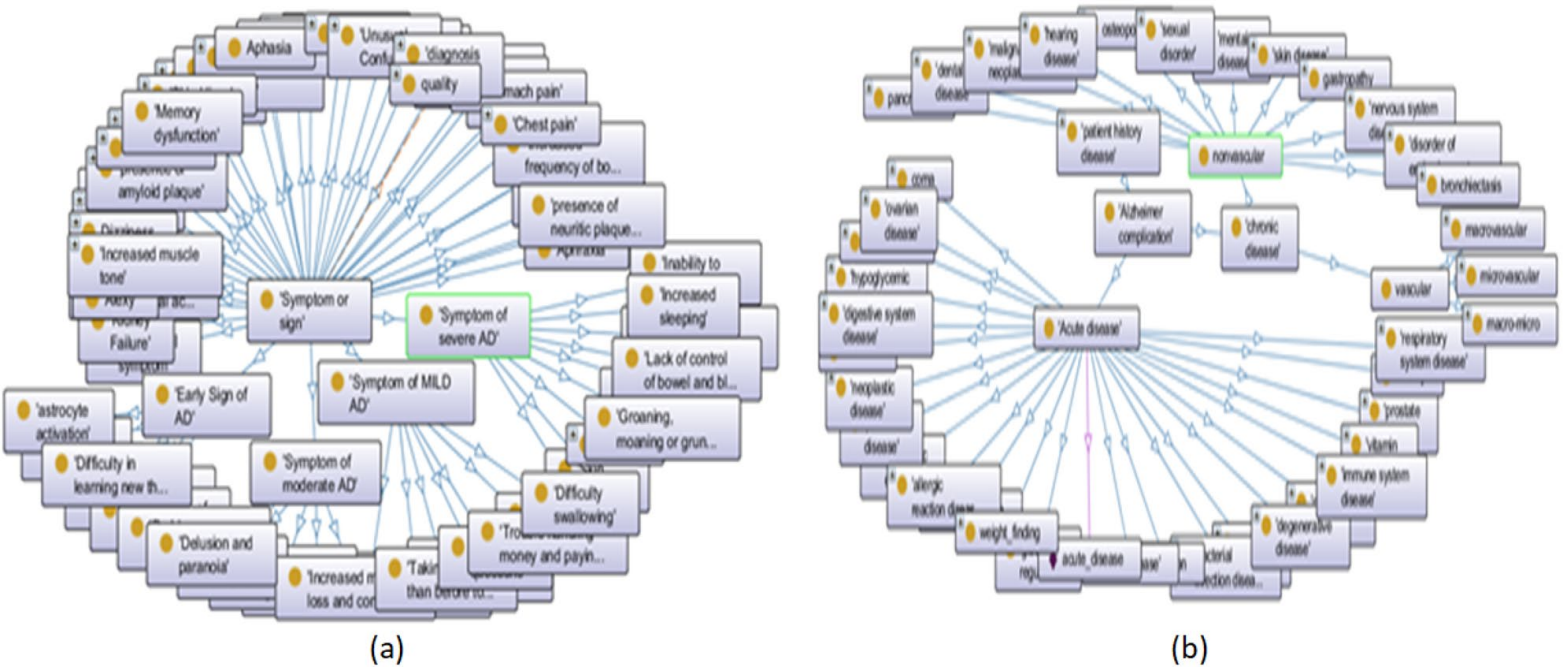
A fragment of the graphical overview of ADDO entities (**a**) related to symptoms concept, (**b**) related to complications concept.

We previously implemented ontology-based knowledge called ADDO^37^. It is important in terms of the standardization, comprehensiveness, and consistency of its interpretable reasoner classifier based on SWRL rule-based reasoning capabilities. It was introduced to access aspects of AD related to the study and diagnosis. It includes patient demographics, medical history, vital signs, medications, and symptoms. In addition, ADDO is supported by various aspects of AD diagnostic tests such as screening tests, neuropsychological assessments, cerebrospinal fluid tests, mood evaluations, cognitive tests, genetics, biological biomarkers, PET scans, and MRI of the brain. Figure 4 shows a fragment graphical overview of ADDO entities related to Symptoms and complications concepts. ADDO is extended to the fuzzy ontology for performing approximate reasoning. But it is not an easy task because

- Ontology editor tools do not provide well-defined means for expression fuzziness. They are mostly based on crisp logic. Its fuzzy extension is usually done using fuzzy OWL 2 Protégé’s plugin based on Fuzzy logic, which is built as a Protégé 3.3.1 tab plug-in.
- It requires more detailed implementation steps like creating fuzzy concepts, roles, Fuzzy modifiers, and fuzzy data types for each Fuzzy term. This means expanding ADDO with new Fuzzy concepts and properties.
- Facing the complexity of ADDO, the consequent negative impact on reasoning time, and the difficulty of handling errors.

In contrast to our previous work on diagnosing AD based on Fuzzy ADDO, this paper has contributions to implement fuzzy approximation reasoning based on crisp ADDO reasoning tasks and fuzzy logic. In fact, ADDO is meaningful knowledge to represent the symptoms and complications of AD and determine their level of severity. Thus, the degree of severity of symptoms and complications, in addition to other diagnostic factors, can lead to Fuzzy logic in the challenge of addressing ambiguity.

### Fuzzy inference

To diagnose AD, the method of fuzzy inference is defined based on Mamdani-type inference. This is because it is intuitive and well-suited to medical diagnosis, where its domain is uncertain, and the diagnosis rules are created or advised by an expert. Fuzzy inference systems aim to manage imprecise knowledge and provide foundations for approximate reasoning. It uses linguistic variables represented as natural language, linguistic modifiers, fuzzy rules, and approximate reasoning.

Mamdani’s inference process involves first defining the knowledge base, including a set of linguistic variables for all input and output variables, and the fuzzy rules. Second, converting a crisp input value to a fuzzy value is performed by the use of the information in the knowledge base. Third, combining the fuzzified inputs according to fuzzy rules to determine the rule strength and define the consequence of the rule by combining the output membership function and the rule strength. Fourth, expect the membership functions for output to be fuzzy sets. Finally, the best representative crisp value of this aggregated output fuzzy set is determined by Defuzzification. The fuzzy results generated cannot be used in an application where the decision has to be taken only on crisp values. Fuzzy inference functional blocks, including linguistic variables, fuzzification, fuzzy rule base, and defuzzification, are illustrated as follows.

#### Linguistic variables

**Figure 5:**
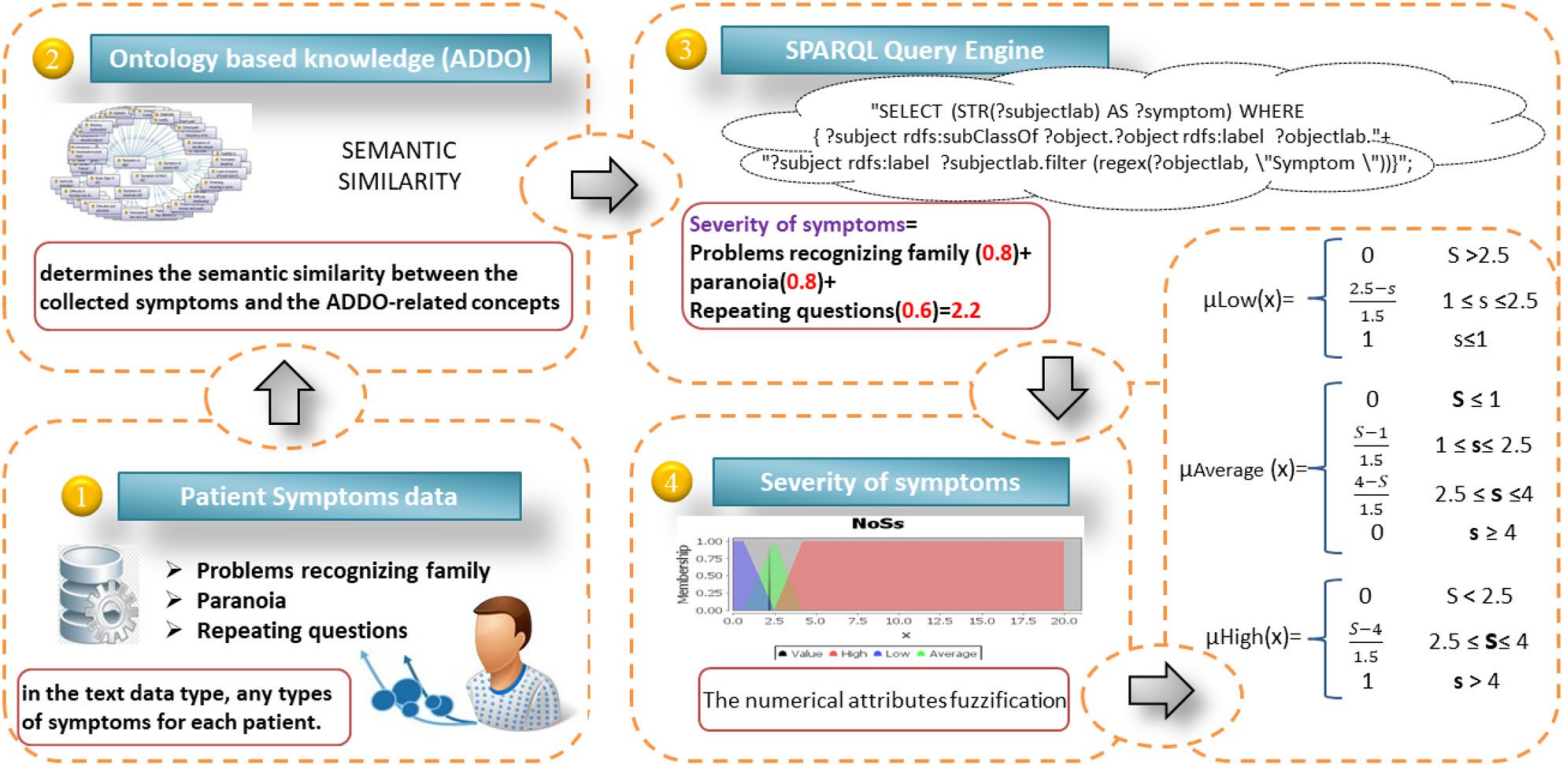
The fuzzification of the severity of symptoms.

**Figure 6:**
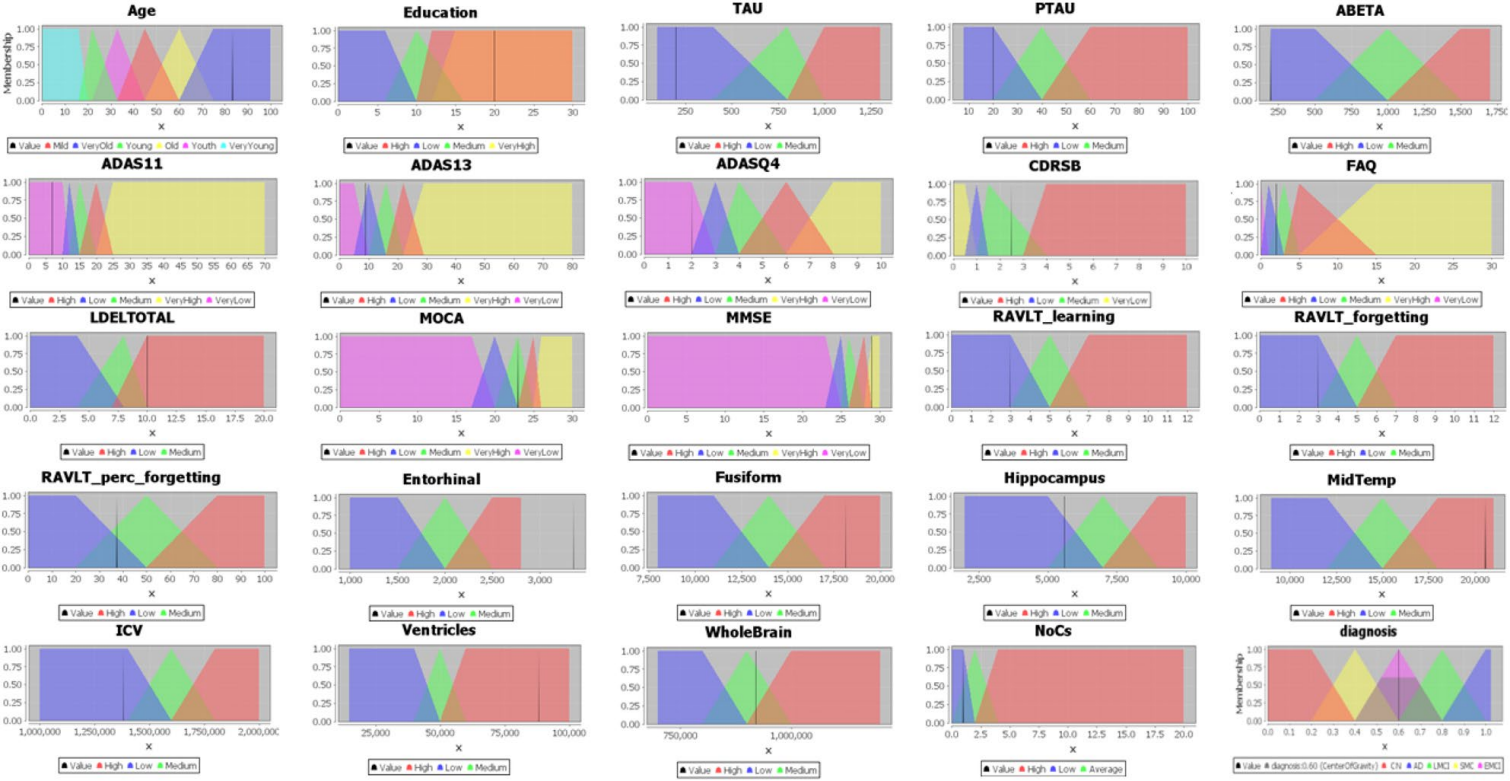
The membership functions for some AD features fuzzy sets. Each fuzzy set spans a region of input (or output) values graphed against membership. For example, for the process of fuzzification of the CDRSB, which is a common cognitive test, there are four fuzzy sets for CDRSB: VeryLow, Low, Medium, and High. The membership function for fuzzy sets VeryLow and High are trapezoidal, while the membership function for Low and Medium is triangular. Any particular input is interpreted from the concerned fuzzy set, and a degree of membership is obtained.

In this section, linguistic variables are defined for the n inputs; for each $j=1,2,\ldots ,n$, a linguistic variable is defined as $V_j$ = $\left( v_j,~ X_j,~ Q_j,~ S_j,~ I_j \right)$, where $v_j$ is the name of the variable; $X_j$ is the domain of the variable; $Q_j$ = $\{q_{j1},~ q_{j2},\ldots , q_{jm}\}$ is a set of m linguistic values for the variable (e.g., Large, Medium, Small); *Sj* = $\{s_{j1},~s_{j2},\ldots , s_{jm}\}$ is a set of fuzzy sets on $X_j$, $s_{jk}$: $X_j$ $\rightarrow$ [0, 1]; $I_j$ associates each linguistic value $q_{jk}$ to a fuzzy set $s_{jk}$.

Different AD features are defined as fuzzy linguistic, including numerical values such as brain volumes data (e.g., Ventricles, Hippocampus, and Fusiform), many cognitive tests (e.g., CDRSB, ADAS13, MMSE, and FAQ), lab tests (e.g., Vitamin B12, Hemoglobin, total cholesterol, and serum uric acid), nonfuzzy ordinal (set up as many singleton membership functions, e.g., APOE4), non-fuzzy semantic (e.g., complications like arterial hypertension, sleep disorder, and skin infection), and non-fuzzy textual (e.g., symptoms). In non-fuzzy textual symptoms, we set up the severity of symptoms in its decision that were identified by the ontology reasoning, as shown in Fig. 5. AD features are fuzzified with the guidance of AD diagnosis clinical practice guidelines and our domain experts. To generate fuzzy partitions, we depend on a number of triangular and trapezoidal, as shown in Fig. 6.

#### Fuzzification

**Figure 7:**
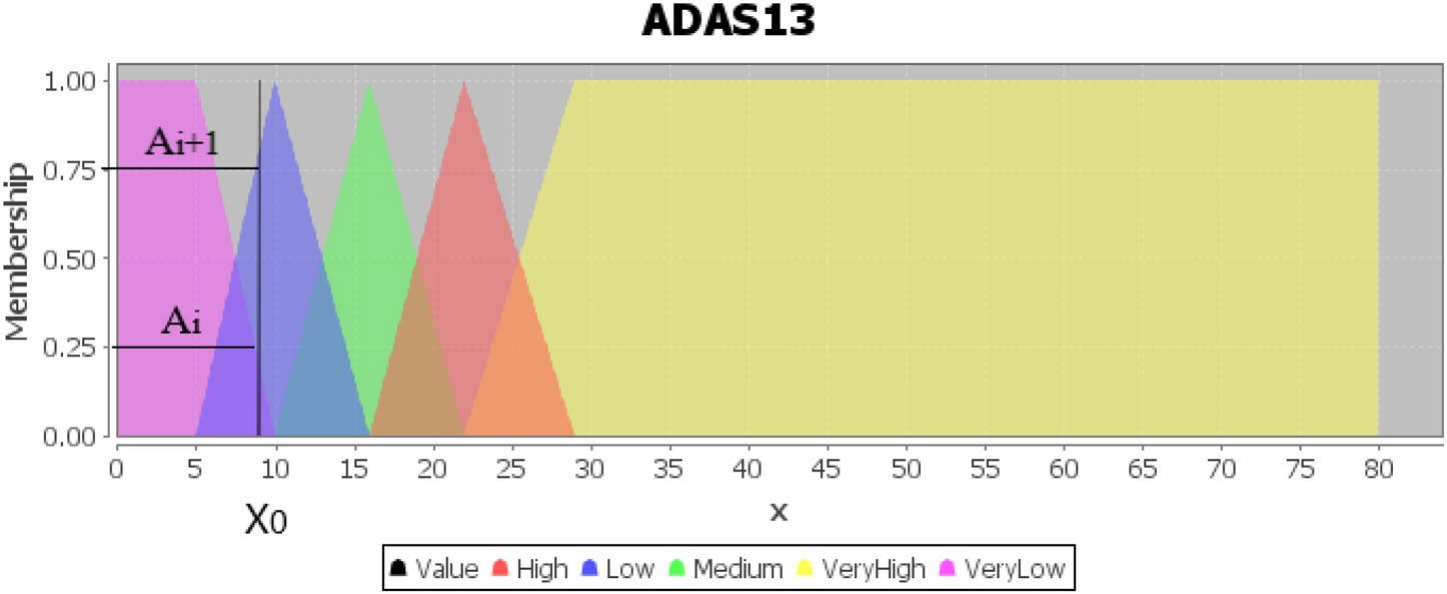
ADAS13 defined by fuzzy set theory.

Through fuzziness, the belonging of all crisp input/output data to the appropriate fuzzy sets expressed in linguistic variables is determined by the membership. This allows rules to be utilized simply. As shown in Fig. 7, fuzzy sets for ADAS13 graphed with trapezoidal and triangular membership functions. There are five fuzzy sets for feature ADAS13: VeryLow, Low, Medium, High, and VeryHigh. Any particular input is interpreted from this fuzzy set, and a degree of membership is obtained. When a patient ADAS13 value is input, there are two fuzzy input values, $\mu A_i(x_0)$ and $\mu A_{i+1}(x_0)$. They can be the truth values of $x_0$ related to $A_i$ and to $A_{i+1}$, respectively. For example, an ADAS13 variable entry with a value of 9 would be as VeryLow as 0.75^∘^ and as low as 0.25^∘^.

#### Fuzzy rule base

**Figure 8:**
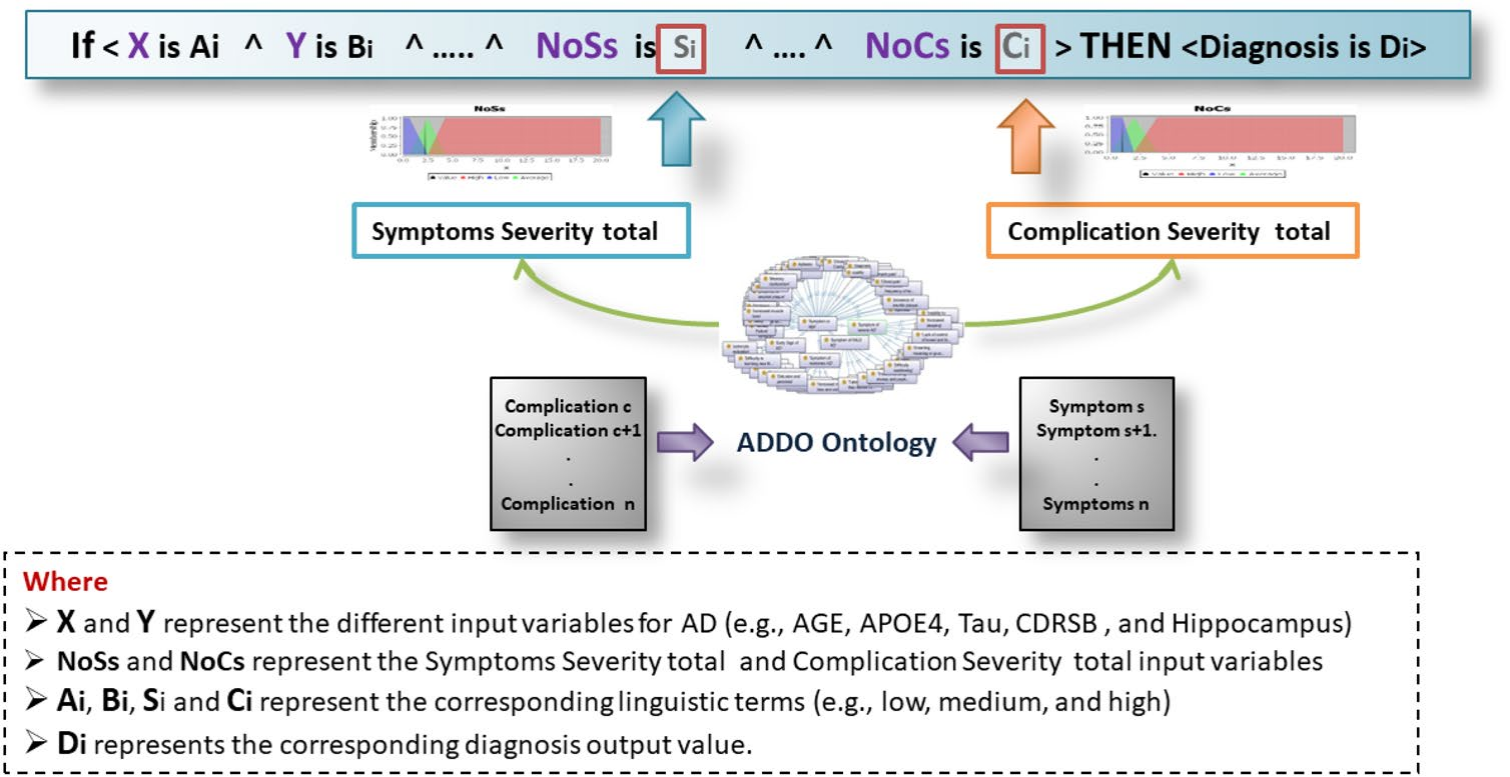
The fuzzy dynamic diagnostic rules with semantic.

In technical medical diagnostic systems, the fundamental problem is determining the appropriate diagnosis for any given set of conditions. Traditional techniques are difficult to rely upon and are usually a set of static rules involving a small number of variables with finite values. The contribution of our methodology is the ability to extend the knowledge base of fuzzy reasoning with semantic capabilities. It’s critical to building medically applicable diagnoses. The knowledge base also includes fuzzy dynamic diagnostic rules, as shown in Fig. 8. Table 2 provides samples of generated fuzzy rules.

**Table 2:** Example of fuzzy rules generated.

Sample of rules	
IF CDRSB IS VeryLow AND FAQ IS VeryLow AND ADAS13 IS VeryLow AND MMSE IS VeryHigh AND MOCA IS VeryHigh AND FDG IS VeryHigh AND S_Severity IS Low AND C_Severity IS Low THEN DR IS CN	
IF CDRSB IS VeryLow AND FAQ IS VeryLow AND ADAS13 IS VeryLow AND MMSE IS VeryHigh AND FDG IS VeryHigh AND S_Severity IS Low AND C_Severity IS Low THEN DR IS CN	
IF CDRSB IS VeryLow AND FAQ IS VeryLow AND ADAS13 IS VeryLow AND MMSE IS VeryHigh AND MOCA IS VeryHigh AND S_Severity IS Low AND C_Severity IS Low THEN DR IS CN	
IF CDRSB IS VeryLow AND Age IS Old AND MOCA IS VeryHigh AND Hippocampus IS High AND S_Severity IS Low AND C_Severity IS Low THEN DR IS CN	
IF CDRSB IS VeryLow AND Age IS Old AND MOCA IS VeryHigh AND Hippocampus IS High AND FDG IS VeryHigh AND RAVLT_immediate IS High AND S_Severity IS Low AND C_Severity IS Low THEN DR IS CN	
IF CDRSB IS VeryLow AND MOCA IS VeryHigh AND FAQ IS Low AND RAVLT_perc_forgetting IS Low AND FDG IS VeryHigh AND S_Severity IS Low AND C_Severity IS Low THEN DR IS CN	
IF CDRSB IS VeryLow AND LDELTOTAL IS High AND S_Severity IS Low AND C_Severity IS Low THEN DR IS CN	
IF CDRSB IS VeryLow and FDG IS High and LDELTOTAL IS High AND S_Severity IS Low AND C_Severity IS Low THEN DR IS SMC	
IF CDRSB IS VeryLow AND MOCA IS High AND S_Severity IS Low AND C_Severity IS Low THEN DR IS SMC	
IF MMSE IS Medium AND LDELTOTAL IS High AND CDRSB IS Low AND S_Severity IS Average AND C_Severity IS Average THEN DR IS EMCI	
IF MMSE IS Medium AND LDELTOTAL IS Medium AND Education IS High AND S_Severity IS Average AND C_Severity IS Average THEN DR IS EMCI	
IF MMSE IS Medium AND LDELTOTAL IS High AND CDRSB IS Low AND MOCA IS VeryHigh AND S_Severity IS Average AND C_Severity IS Average THEN DR IS EMCI	
IF CDRSB IS Medium AND FAQ IS High AND LDELTOTAL IS Medium AND S_Severity IS Average AND C_Severity IS Average THEN DR IS EMCI	
IF CDRSB IS Medium AND FAQ IS High AND LDELTOTAL IS Low AND S_Severity IS Average AND C_Severity IS Average THEN DR IS LMCI	
IF CDRSB IS Medium AND FAQ IS High AND LDELTOTAL IS Medium AND Education IS VeryHigh AND S_Severity IS Average AND C_Severity IS Average THEN DR IS LMCI	
IF FAQ IS VeryHigh AND CDRSB IS High AND S_Severity IS High AND C_Severity IS High THEN DR IS AD	
IF FAQ IS VeryHigh AND MMSE IS VeryLow AND S_Severity IS High AND C_Severity IS High THEN DR IS AD	
IF FAQ IS VeryHigh AND MOCA IS Low AND S_Severity IS High AND C_Severity IS High THEN DR IS AD	

The min-max Mamdani technique was used to infer the premise of the rule. It is based on using the max t-conorm operator for aggregation. On the other hand, the min t-norm operator is used for conjunction (AND) and for the implication function.

#### Defuzzification

**Figure 9:**
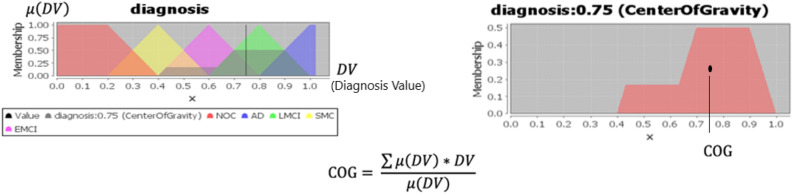
The output membership function.

In general, real-world applications require crisp values other than fuzzy inference results, which is a fuzzy set. This is where defuzzification is important in mapping a fuzzy space into a non-fuzzy. One of the most common methods is the center of gravity (COG) which takes the weighted average of the area bounded by the membership function curve as the crisp value of the fuzzy quantity. The defuzzification technique in which the fuzzy data are represented by the coordinates of the COG of the level’s section contained between the graph of the membership function involved and the X-axis. According to Fig. 9, the defuzzification has been done using the output membership function. The result of the defuzzification process for the Diagnosis Value of a patient (p) is a crisp value that combines evidence from the considered input parameters [e.g., patient (p) features] and accordingly indicates the person’s Diagnosis Value (DV), which is denoted DV(p).

### System implementation

**Figure 10:**
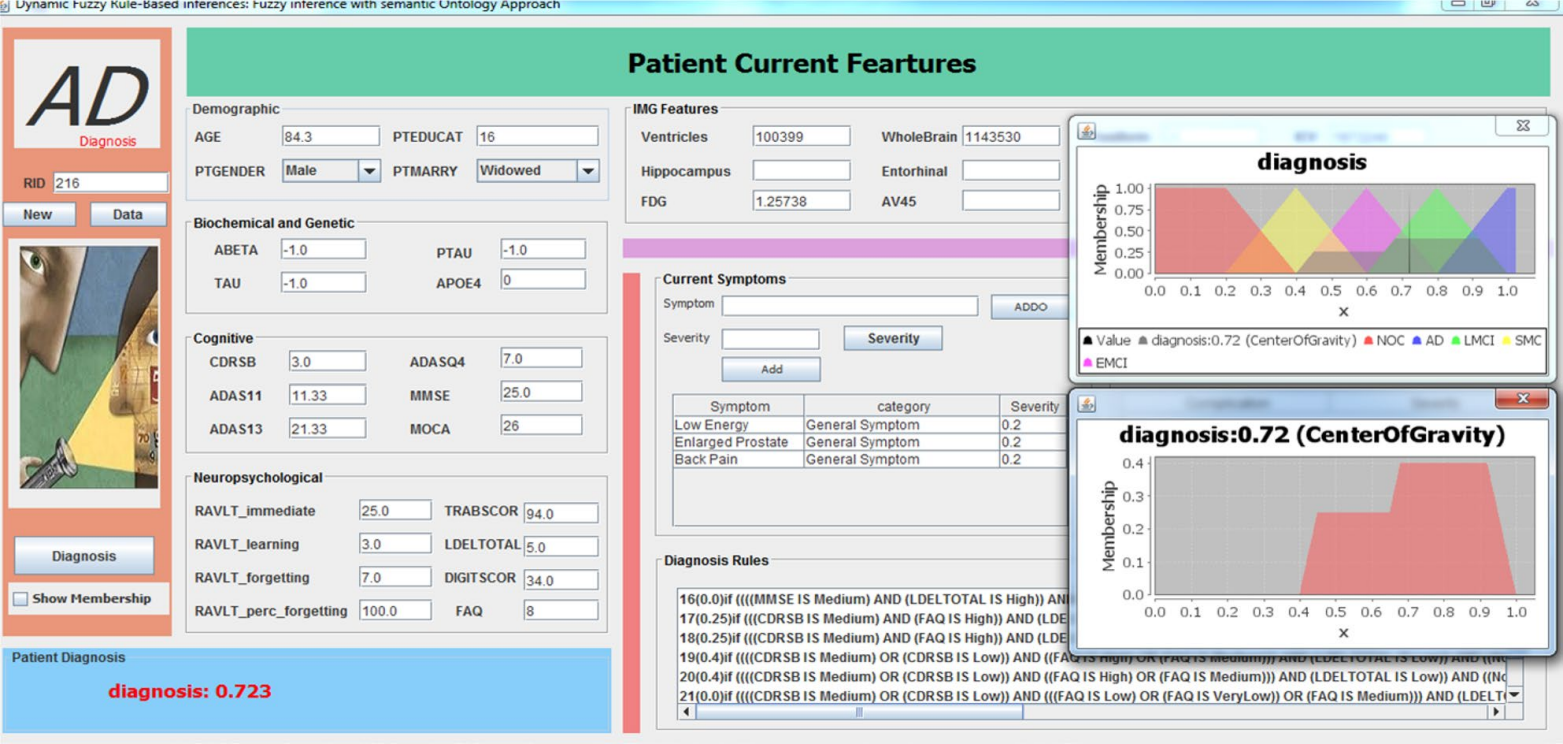
The interface for the physician to input patient characteristics. The interface supports two ways to handle semantic features. The first way is to click the ADDO button, where a list is displayed containing symptoms or complications of ADDO. The clinicians can define any number of symptoms for each patient, and ADDO ontology determines the severity of each specific symptom. The second way is that clinicians can manually enter the symptoms. Furthermore, clinicians can determine the severity of each symptom, which supports customized decision-making. The same steps are done for complications. The inference provides a correct diagnosis result as LMCI for an ADNI subject with the real patient case RID 216 (recorded in ADNI dataset).

In this paper, a DFRI system is presented to provide accurate and semantically interpretable AD diagnosis. It utilizes AD knowledge and Mamdani by adding semantic features in the form of ontology to fuzzy rule-based inferences. It makes the fuzzy inferences system more accurate and deals with semantic features easier as well as reuses AD knowledge among different systems. The implementation is based mainly on Jena JAVA and jFuzzyLogic APIs^49^. The development environment is Eclipse IDE Version: 2021-03 (4.19.0) with JDK jdk-15.0.2. We utilized our ADDO ontology for AD symptoms and complications sources and Weka5 for learning purposes. Based on the DFRIs GUI, patient data can be edited or displayed from the dataset. In addition, a list containing symptoms and/or complications that are stored in ADDO is displayed by clicking the ADDO button. The Diagnose button executes the fuzzy inference process, as shown in Fig. 10.

## Experiment and results

This section presents the results for the proposed DFRIs system using the ADNI data set. we deal efficiently with the high dimensionality nature of the ADNI. We considered 68 numerical features associated with AD [demographics (2), vital signs (2), genetics (1), cognitive scores (12), lab tests (42), MRI (7), and PET (2)], in addition to a very large number of symptoms decreased according to the different stages of AD. Table3 shows a sample of symptoms, as well as complications, in the form of semantic data stored in ADDO ontology.

**Figure 11:**
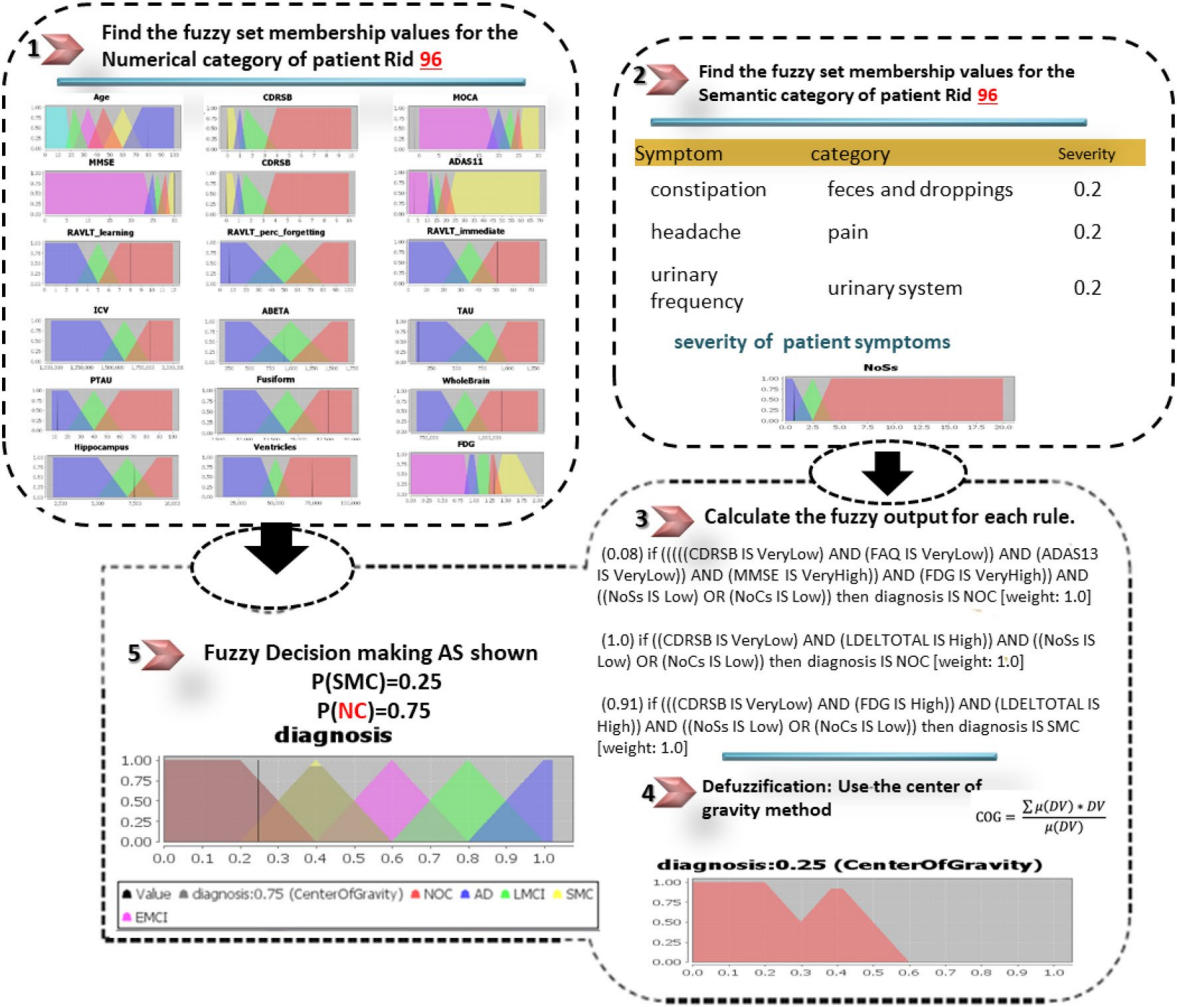
A correct diagnosis result of the proposed DFRIs as CN for an ADNI patient with RID 96; a male patient; has a real diagnosis of CN; aged 79.6; has symptoms of (constipation, headache, urinary frequency).

**Figure 12:**
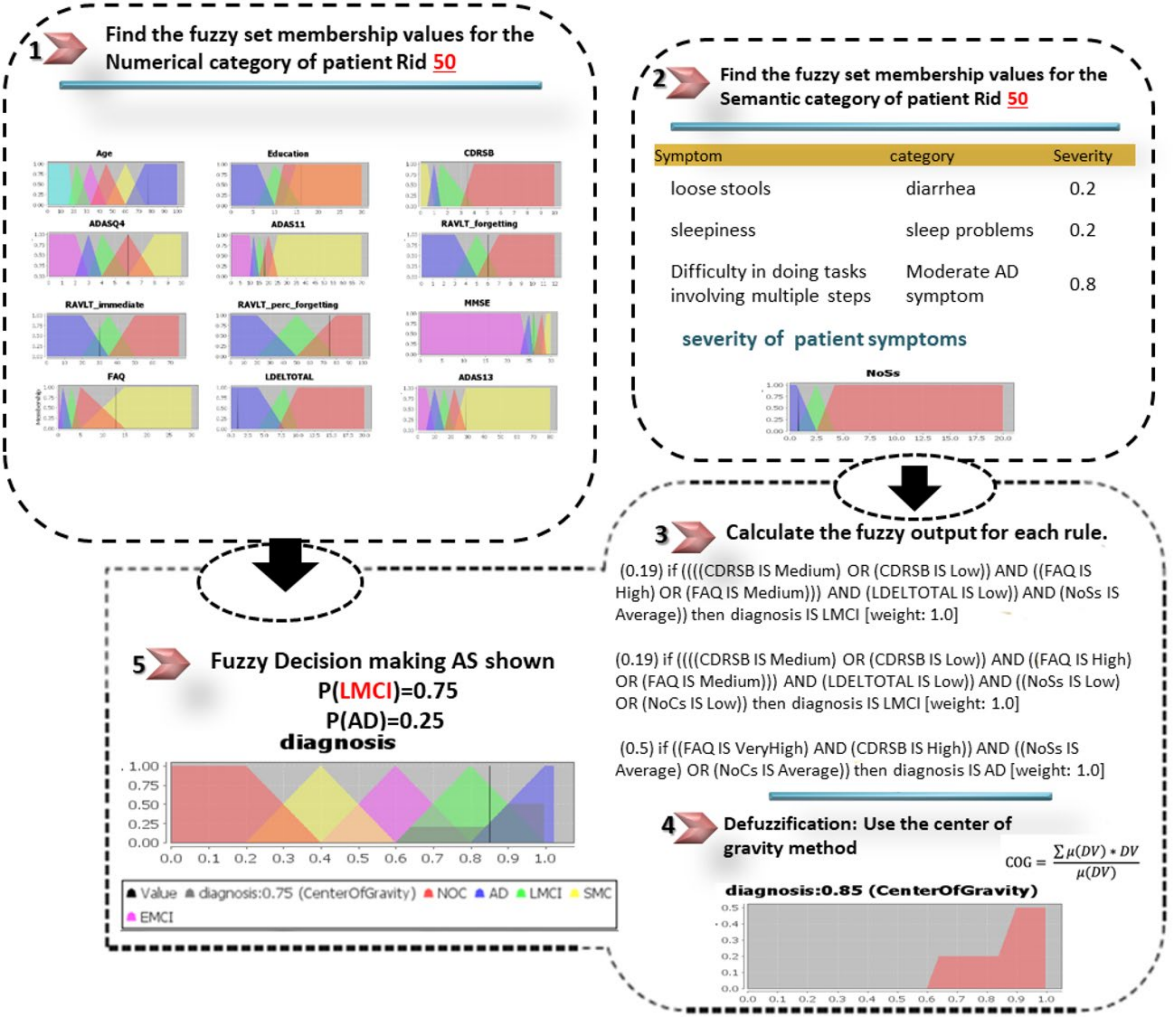
A correct diagnosis result of the proposed DFRIs as LMCI for an ADNI patient with RID 50; a male patient; has a real diagnosis of LMCI; aged 77.6; has symptoms of (loose stools, sleepiness, Difficulty in doing tasks involving multiple steps).

**Figure 13:**
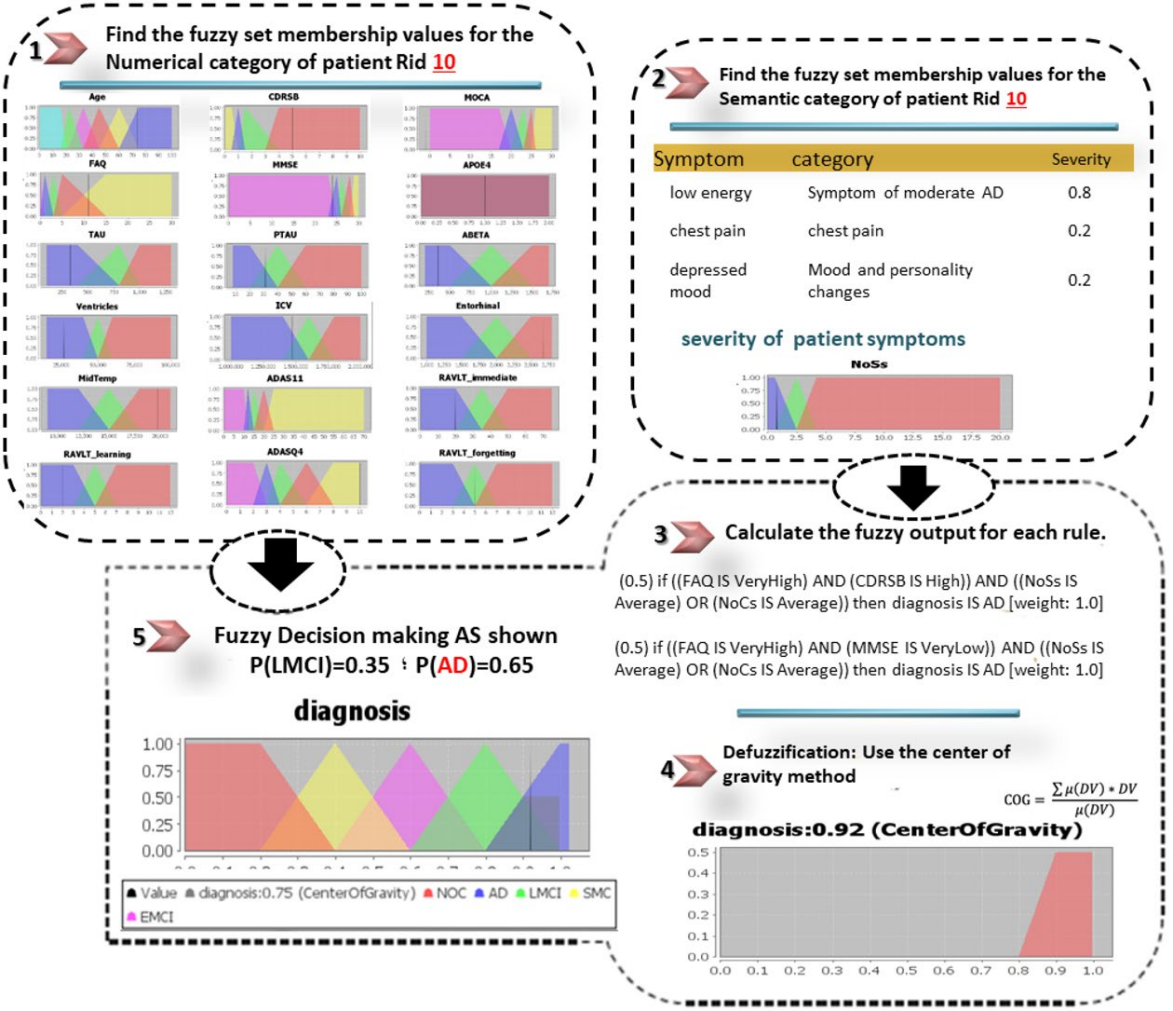
A correct diagnosis result of the proposed DFRIs as AD for an ADNI patient with RID 10; a female patient; has a real diagnosis of AD; aged 73.9; has symptoms of (low energy, chest pain, depressed mood).

The DFRIs system is evaluated using 30 CN, 35 SMC, 56 EMCI, 60 LMCI, and 50 AD samples. The classification results achieved 97.2%, 95.4%, 94.8%, 93.1%, and 96.3% accuracy results for AD, LMCI, EMCI, SMC, and CN, respectively. Based on the GUI proposed, we offer three case studies, as follows; see Figs. 11, 12 and 13.

**Table 3:** Example of symptoms.

Sample of symptoms	
Dry mouth; light sweating; urinary frequency; blood sugar low; knee pain; intermittent rash; ankle swelling; mild shortness of breath; knee stiffness; falls; diarrhea; low energy; feels drowsy; short of breath; palpitations; low night vision; rapid heartbeat; arthritis; loose stools; topical dermatitis; winded walking up stairs; knee stiffness; occasional crying; episodic unsteadiness; can’t sleep; depressive symptoms; bilateral loss of hearing; ankle swelling; difficulty falling asleep; arms elbows and knees; decreased energy for walking; sleep problems; musculoskeletal pain; blurred vision; unable to pass bowel; trouble breathing; depressed mood; constipation; dizziness; mild discomfort; low energy; insomnia; nighttime perspiration	

### Comparison with other ontology-based systems

Table 4 presents the comparison of the proposed DFRIs system in terms of ontology reasoning performance with existing AD diagnosis ontology based on the ADNI dataset. Although the ontology-driven decision support system achieved slightly better results, it is only limited to distinguishing MCI patients from CN based solely on MRI data. The proposed system outperformed the majority of the existing techniques. Our proposed method obtained the most significant results in terms of dealing with the high dimensionality nature of the ADNI especially semantic data as symptoms and complications. By using the proposed DFRIs system, we do not have to enter all patient data into the ontology, which takes time and great effort and whose errors are difficult to detect and treat. The role of ontology is limited to determining the severity of the semantic data specific to each patient and then entering them as numerical input to the fuzzy logic. Additionally, our proposed model DFRIs is comparably better for dealing with fuzzification and medical linguistic terms than existing machine learning techniques shown in Table 5.

### Comparison with other machine learning systems

**Figure 14:**
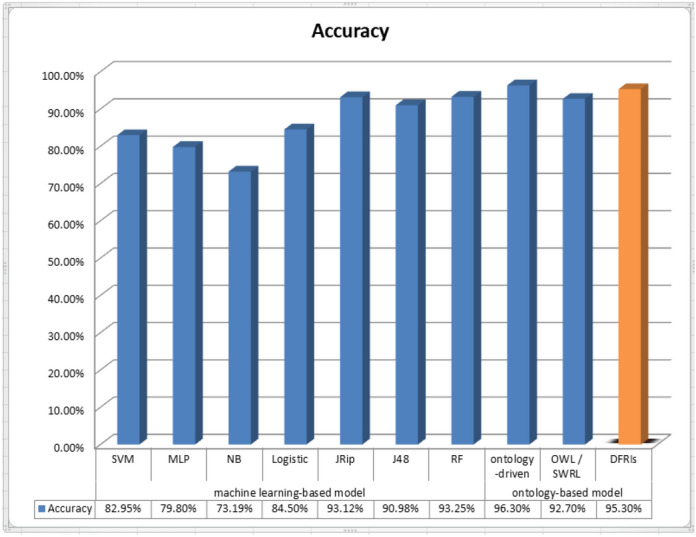
Comparison of different classifiers (ontology-based; machine learning based), where our system DFRIs yields the best predictive performance. These accuracy score results were obtained as follows. Firstly for the learning techniques, this evaluation was conducted by us using the same ADNI dataset including SVM, MLP, NaiveBayes, SimpleLogistic, JRip, J48, RandomForest, and our proposed DFRIs. Secondly, for the ontology-based diagnosis systems^33,36^, the accuracy score values were taken from the relevant references.

We also compared DFRIs in terms of accuracy with the existing machine learning techniques for the AD diagnosis based on the ADNI dataset, which is presented in Table 5. DFRIs attained a score of 95.3% and outperformed the other existing approaches. We avoided the traditional methods of dealing with missing data, knowing that the ADNI dataset suffers from missing data. However, this missing can be considered normal because the requirements for medical diagnosis are variable, and there are many alternatives. For example, there are a large number of cognitive tests, each with its own characteristics. It is not normal for the patient to conduct all these tests together. Therefore, we find that the patient record contains null values for some tests. Sometimes, some tests are common and easier for the patient and enjoy reasonable prices, as is the case with MRI and FDG, where the FDG suffers from more null values than the MRI. DFRIs system is a knowledge-based decision system that is more flexible in dealing with medicinal nature without having to fill in the null values. It also has an explanation feature, not a black box model, which is useful for new doctors with little experience; the proposed DFRIs system provides an interpretation of how it reached the diagnosis decision. Additionally, the DFRIs system is comparably fuzzy and flexible for dealing with semantic data as symptoms. Without adhering to certain symptoms with specific values, the DFRIs system has a dynamic character. A comparison of the accuracy results obtained from the ontology-based, machine learning, and proposed DFRIs is shown in Fig. 14.

**Table 4:** Analysis of the AD diagnosis results in terms of the ontology-based model using the ADNI dataset.

	Modalities	Datasets	CN	SMC	EMCI	LMCI	AD
Ontology-driven dss^33^	MRI data	ADNI (364)187 MCI + 177 normal	Distinguish MCI patients from CN with accuracy 96.3%				
OWL/SWRL reasoning^36^	MRI data+ PET+ Genetics+ Cognitive + functional assessments	ADNI (208) 40 CN+ 45 SMC+ 50 EMCI+41 LMCI+ 32 AD	92%	91.3%	94.6%	93.4%	92.6%
DFRIs	MRI data+ PET+ Genetics+ Cognitive + functional assessments+ symptoms+ complications	ADNI (2268) 518 CN+ 313 SMC+ 389 EMCI+651 LMCI+ 397 AD	96.3%	93.1%	94.8%	95.4%	97.2%

The proposed DFRIs performed better in terms of dealing with many AD Modalities, especially semantic data as symptoms and complications. Additionally, DFRIs system is comparably good for dealing with fuzzification.

**Table 5:** Analysis of the AD diagnosis results in terms of the machine learning techniques using the ADNI dataset.

	Accuracy (%)	Precision	Recall	F-Measure
		(AD, CN, EMCI, LMCI, SMC)	(AD, CN, EMCI, LMCI, SMC)	(AD, CN, EMCI, LMCI, SMC)
SVM	82.95	(92, 71, 85, 89, 72)%	(89, 93, 89, 90, 29)%	(90, 81, 87, 90, 42)%
MLP	79.8	(86, 74, 86, 86, 56)%	(88, 76, 84, 86, 55)%	(87, 75, 85, 86, 55)%
NaiveBayes	73.19	(82, 73, 67, 76, 56)%	(88, 84, 71, 69, 42)%	(85, 79, 69, 73, 48)%
SimpleLogistic	84.5	(92, 76, 89, 89, 69)%	(88, 87, 91, 91, 51)%	(90, 81, 90, 90, 59)%
JRip	93.12	(93, 88, 93, 95, 94)%	(95, 96, 94, 93, 80)%	(94, 92, 94, 94, 87)%
J48	90.98	(92, 88, 93, 94, 83)%	(94, 91, 91, 93, 78)%	(93, 90, 92, 94, 80)%
RandomForest	93.25	(91, 89, 94, 95, 96)%	(97, 98, 93, 93, 79)%	(94, 93, 94, 94, 87)%

Compared to existing machine learning techniques shown in this table, the proposed DFRIs performed better in terms of accuracy, dealing with missing data, and dealing with many AD Modalities, especially semantic data as symptoms and complications. Additionally, DFRIs is comparably good for dealing with fuzzification.

## Conclusion

AD is one of the most serious diseases that cause damage to brain cells. As it progresses, brain tissue shrinks and loses its ability to function. The early detection system for AD is a major challenge for many researchers. We attempted to discuss the technical aspects of the current AD diagnosis. MRI criteria are the basis for many AD diagnoses. It is not sensitive enough to be considered a reference criterion in the diagnosis of early AD even when combined with any of the available neuropsychological tests. Particularly, some criteria such as symptoms and complications may be more sensitive to early detection of AD but need specific treatment which varies greatly for each patient, since they have a great relationship with semantics and have very rich linguistic information. In this paper, in contrast to previous similar AD diagnosis systems, fuzzy reasoning with a semantic ontology approach is proposed and implemented. It is characterized by the ability to deal with comprehensive diagnostic categories of AD with varying natures of their data types (numerical, category, and semantic) and relying on inferences based on fuzzy dynamic rules.

This is a novel idea to improve the capabilities of existing fuzzy systems by combining them with the semantic reasoning of ontology. It consists of explicitly defining the semantics of AD knowledge using ontology and dealing with the imprecise and ambiguous nature of its data using fuzzy set theory. We develop and implement an automated diagnosis of AD. The validation of our proposed system is done using real ADNI cases. The resulting system is accurate, interpretable, and dynamic.

In future work, food recommendations for AD patients will be a great challenge. It’s about providing their caregivers with the tools and information they need to make informed decisions about AD patient diets and improve their health outcomes. Ontology can represent semantic knowledge regarding units of measure, side effects, and allergies to diets. This will have a significant impact on enhancing the performance of the system as a whole.

## Data Availability

The datasets generated and analyzed during the current study are available on the ADNI website (http://adni.loni.usc.edu/).

## References

[1] Möller H-J, Graeber MB (1998). The case described by Alois Alzheimer in 1911: Historical and conceptual perspectives based on the clinical record and neurohistological sections. Eur. Arch. Psychiatry Clin. Neurosci..

[2] Harper, L. C. 2022 Alzheimer’s association facts and figures. https. Tech. Rep. (2023). Accessed March 2023.

[3] Kim C (2022). Distinct populations of highly potent TAU seed conformers in rapidly progressing Alzheimer’s disease. Sci. Transl. Med..

[4] Cammisuli DM, Cipriani G, Castelnuovo G (2022). Technological solutions for diagnosis, management and treatment of Alzheimer’s disease-related symptoms: A structured review of the recent scientific literature. Int. J. Environ. Res. Public Health.

[5] Hazan S (2020). Rapid improvement in Alzheimer’s disease symptoms following fecal microbiota transplantation: A case report. J. Int. Med. Res..

[6] Vaz M, Silvestre S (2020). Alzheimer’s disease: Recent treatment strategies. Eur. J. Pharmacol..

[7] Ossenkoppele R (2022). Research criteria for the behavioral variant of Alzheimer disease. JAMA Neurol..

[8] Al-Sarayrah S, Abulail D, Shaalan K (2022). Understanding the impact of the ontology of semantic web in knowledge representation: A systematic review. Recent Innov. Artif. Intell. Smart Appl..

[9] El Massari H (2022). An ontological model based on machine learning for predicting breast cancer. Int. J. Adv. Comput. Sci. Appl..

[10] Bao, Q., Zhao, G., Yu, Y. & Dai, S. Ontology-based assembly process modeling with element extraction and reasoning. In *CAD’21 Proceedings*. 10.14733/cadconfp.2021.1-5 (CAD Solutions LLC, 2021).

[11] Gomez-Valadés, A., Martínez-Tomás, R. & Rincón-Zamorano, M. Ontologies for early detection of the alzheimer disease and other neurodegenerative diseases. In *International Work-Conference on the Interplay Between Natural and Artificial Computation*, 42–50 (Springer, 2019).

[12] Shoaip, N., Barakat, S. & Elmogy, M. Alzheimer’s disease integrated ontology (adio). In *2019 14th International Conference on Computer Engineering and Systems (ICCES)*, 374–379 (IEEE, 2019).

[13] Shoaip N, Sappagh SE, Barakat S, Elmogy M (2020). A framework for disease diagnosis based on fuzzy semantic ontology approach. Int. J. Med. Eng. Inform..

[14] Ghorbani A, Davoodi F, Zamanifar K (2023). Using type-2 fuzzy ontology to improve semantic interoperability for healthcare and diagnosis of depression. Artif. Intell. Med..

[15] El-Sappagh S, Ali F (2016). Ddo: A diabetes mellitus diagnosis ontology. Applied Informatics.

[16] Otte JN, Beverley J, Ruttenberg A (2022). BFO: Basic formal ontology1. Appl. Ontol..

[17] El-Sappagh S, Franda F, Ali F, Kwak K-S (2018). Snomed CT standard ontology based on the ontology for general medical science. BMC Med. Inform. Decis. Mak..

[18] Cross, V. & Chen, S. Fuzzy ontologies: State of the art revisited. In *North American Fuzzy Information Processing Society Annual Conference*, 230–242 (Springer, 2018).

[19] El-Sappagh S, Elmogy M (2017). A fuzzy ontology modeling for case base knowledge in diabetes mellitus domain. Eng. Sci. Technol. Int. J..

[20] Zhai, J., Li, M. & Zhou, K. Linguistic variable ontology and its application to fuzzy semantic retrieval. In *International Conference on Information Computing and Applications*, 188–195 (Springer, 2010).

[21] Ghorbel, H., Bahri, A. & Bouaziz, R. Fuzzy ontologies building method: Fuzzy ontomethodology. In *2010 Annual Meeting of the North American Fuzzy Information Processing Society*. 10.1109/nafips.2010.5548211 (IEEE, 2010).

[22] Ktistakis IP, Goodman G, Shimizu C (2021). Methods for optimizing fuzzy inference systems. Advances in Data Science: Methodologies and Applications.

[23] Bobillo, F. & Straccia, U. Representing fuzzy ontologies in owl 2. In *International Conference on Fuzzy Systems*, 1–6 (IEEE, 2010).

[24] Borgwardt S, Distel F, Peñaloza R (2015). The limits of decidability in fuzzy description logics with general concept inclusions. Artif. Intell..

[25] Zekri F, Ellouze AS, Bouaziz R (2020). A fuzzy-based customisation of healthcare knowledge to support clinical domestic decisions for chronically ill patients. J. Inf. Knowl. Manage..

[26] Samhan LF, Alfarra AH, Abu-Naser SS (2022). Classification of Alzheimer’s disease using convolutional neural networks. Int. J. Acad. Inf. Syst. Res..

[27] Chui KT, Gupta BB, Alhalabi W, Alzahrani FS (2022). An MRI scans-based Alzheimer’s disease detection via convolutional neural network and transfer learning. Diagnostics.

[28] Liu J (2021). Alzheimer’s disease detection using depthwise separable convolutional neural networks. Comput. Methods Programs Biomed..

[29] Savaş S (2022). Detecting the stages of Alzheimer’s disease with pre-trained deep learning architectures. Arab. J. Sci. Eng..

[30] El-Sappagh S, Abuhmed T, Islam SR, Kwak KS (2020). Multimodal multitask deep learning model for Alzheimer’s disease progression detection based on time series data. Neurocomputing.

[31] El-Sappagh S (2021). Alzheimer’s disease progression detection model based on an early fusion of cost-effective multimodal data. Futur. Gener. Comput. Syst..

[32] Sanchez, E. *et al.* A knowledge-based clinical decision support system for the diagnosis of alzheimer disease. In *2011 IEEE 13th International Conference on e-Health Networking, Applications and Services*, 351–357 (IEEE, 2011).

[33] Zhang X, Hu B, Ma X, Moore P, Chen J (2014). Ontology driven decision support for the diagnosis of mild cognitive impairment. Comput. Methods Programs Biomed..

[34] Ivascu, T., Manate, B. & Negru, V. A multi-agent architecture for ontology-based diagnosis of mental disorders. In *2015 17th International Symposium on Symbolic and Numeric Algorithms for Scientific Computing (SYNASC)*, 423–430 (IEEE, 2015).

[35] Toro C (2012). Using set of experience knowledge structure to extend a rule set of clinical decision support system for Alzheimer’s disease diagnosis. Cybern. Syst..

[36] Shoaip N (2021). Alzheimer’s disease diagnosis based on a semantic rule-based modeling and reasoning approach. Comput. Mater. Contin..

[37] Shoaip N (2020). A comprehensive fuzzy ontology-based decision support system for Alzheimer’s disease diagnosis. IEEE Access.

[38] Bangyal WH (2022). Constructing domain ontology for Alzheimer disease using deep learning based approach. Electronics.

[39] Benson GS (2022). Don’t forget about tau: The effects of apoe4 genotype on Alzheimer’s disease cerebrospinal fluid biomarkers in subjects with mild cognitive impairment-data from the dementia competence network. J. Neural Transm..

[40] Cersonsky TE (2022). Using the Montreal cognitive assessment to identify individuals with subtle cognitive decline. Neuropsychology.

[41] Huang H-C, Tseng Y-M, Chen Y-C, Chen P-Y, Chiu H-Y (2021). Diagnostic accuracy of the clinical dementia rating scale for detecting mild cognitive impairment and dementia: A bivariate meta-analysis. Int. J. Geriatr. Psychiatry.

[42] Arevalo-Rodriguez I (2021). Mini-mental state examination (MMSE) for the early detection of dementia in people with mild cognitive impairment (MCI). Cochrane Database Syst. Rev..

[43] van Loenhoud AC (2019). Cognitive reserve and clinical progression in Alzheimer disease: A paradoxical relationship. Neurology.

[44] Andrews JS (2019). Disease severity and minimal clinically important differences in clinical outcome assessments for Alzheimer’s disease clinical trials. Alzheimer’s Dementia Transl. Res. Clin. Interv..

[45] Putcha D (2019). Fractionating the rey auditory verbal learning test: Distinct roles of large-scale cortical networks in prodromal Alzheimer’s disease. Neuropsychologia.

[46] Fokuoh E (2021). Longitudinal analysis of apoe-ε4 genotype with the logical memory delayed recall score in Alzheimer’s disease. J. Genet..

[47] Eroglu Y, Yildirim M, Cinar A (2022). MRMR-based hybrid convolutional neural network model for classification of Alzheimer’s disease on brain magnetic resonance images. Int. J. Imaging Syst. Technol..

[48] Yang Z, Liu Z (2020). The risk prediction of Alzheimer’s disease based on the deep learning model of brain 18f-fdg positron emission tomography. Saudi J. Biol. Sci..

[49] Cingolani P, Alcalá-Fdez J (2013). jfuzzylogic: A java library to design fuzzy logic controllers according to the standard for fuzzy control programming. Int. J. Comput. Intell. Syst..

